# Soluble Dietary Fiber of Hawthorn Relieves Constipation Induced by Loperamide Hydrochloride by Improving Intestinal Flora and Inflammation, Thereby Regulating the Aquaporin Ion Pathway in Mice

**DOI:** 10.3390/foods13142220

**Published:** 2024-07-15

**Authors:** Henghui Zhang, Qixin Zu, Jiancai Zhang, Suwen Liu, Guohua Zhang, Xuedong Chang, Xiaojun Li

**Affiliations:** 1 Department of Environment and Safety Engineering, Taiyuan Institute of Technology, Taiyuan 030008, China; 2 College of Food Science & Technology, Hebei Yanshan Special Industrial Technology Research Institute, Hebei Normal University of Science and Technology, Qinhuangdao 066004, China; 3 School of Life Science, Shanxi University, Taiyuan 030006, China; 4 School of Chemical Engineering and Technology, North University of China, Taiyuan 030051, China

**Keywords:** soluble dietary fiber, constipation, intestinal flora, gastrointestinal hormones, aquaporin

## Abstract

Dietary fiber can be fermented and utilized by gut microbiota to reshape the gut microbiota, thereby alleviating constipation. This experiment mainly studied the physicochemical functions of hawthorn soluble dietary fiber (HSDF)and its effect and mechanism in alleviating constipation in mice. Forty-five mice were divided into blank control group C, model group M, positive control HS group, low-dose LHSDF group (1 g/kg/bw), and high-dose HHSDF group (2 g/kg/bw). The mice were modeled at a dose of 10 mg/kg/bw of loperamide hydrochloride for 7 days, while the remaining groups were orally administered an equal amount of distilled water and test samples. After continuous gavage for 45 days we performed a bowel movement test, and then continued gavage for 7 days and performed a small intestine propulsion test and indicator testing. The results showed that HSDF is mainly composed of galacturonic acid, belonging to the type I crystal structure, with a loose surface resembling a snowflake, a small molecular weight, and strong water-holding and antioxidant abilities. Animal experiments showed that compared with group M, HSDF significantly upregulated AQP3 and AQP8 by 52.67% and 164.54%, respectively, and downregulated AQP9 protein expression by 45.88%, thereby promoting intestinal peristalsis. It can also alleviate constipation by increasing the levels of excitatory hormones such as MTL, GAS, and SP in the gastrointestinal tract, and reducing the levels of inhibitory hormones such as SS, NO, and MDA. In addition, HSDF can reduce the levels of inflammatory factors such as IL-6 and PL-1 β, increase the content of various short-chain fatty acids, alleviate intestinal inflammation, maintain intestinal integrity, and promote defecation. It can also promote the growth of probiotics such as Bacteroides, inhibit the growth of harmful bacteria such as Bifidobacterium and Lactobacillus, and alter the diversity of gut microbiota.

## 1. Introduction

Constipation is a common, complex symptom in digestive medicine. It is characterized by reduced defecation frequency and volume, dry defecation, and laborious defecation [1], often accompanied by abdominal distension, vomiting, restlessness, and colon obstruction in severe cases [2]. General pathological causes of constipation include enteric nervous system dysfunction, visceral hypersensitivity, abnormal distribution of interstitial Cajal cells, and low gastrointestinal motility [3]. Functional constipation, which manifests as hard and relatively little stool but no organic or structural diseases, is the most common [4]. Presently, the global prevalence of constipation is as high as 20%. Furthermore, psychological stress and dietary or nutritional problems are the main causal factors of functional constipation [5], with intestinal inflammation and microecological imbalance being significant pathogenic factors [6]. Nowadays, most people treat constipation using drugs or by changing their lifestyle. However, the use of drugs for treatment has more side effects, and long-term use causes additional problems such as intestinal nerve damage, malnutrition, and aggravation of constipation symptoms after drug discontinuation [7,8]. Therefore, the identification and development of natural active ingredients of foods that relieve constipation has extensive application prospects.

Foods with prebiotic function can relieve or improve symptoms of constipation [9]; the natural carbohydrates of numerous plants and macrofungi have been found to have potential constipation relief effects [10]. Foods rich in soluble dietary fiber (SDF) are beneficial for the improvement of constipation [11], and a high-fiber diet was found to promote fecal excretion and to shorten colonic transit time [12]. Dietary fiber can also increase the content of beneficial bacteria such as Bacteroidales, Erysipelotrichaceae, Ruminococcus, and Akkermansia in the intestinal tract [13]. The beneficial bacteria can increase the content of SCFAs in the intestinal tract, maintain intestinal pH, protect intestinal health, and alleviate symptoms of constipation [14].

Constipation is closely associated with aquaporins (AQPs) in the colon and gastrointestinal hormones. AQP3, AQP8, and AQP9 are intestinal expression proteins that play an important role in the transmembrane transport of intestinal water [15]. Studies have found that abnormal gastrointestinal hormones are important causal factors of constipation. Gastrointestinal hormones are mainly secreted by chemical messenger cells of the gastrointestinal mucosa, belonging to the intestinal neuroendocrine system and essential substances in the intestinal tract [1]. Both aquaporins and gastrointestinal hormones are affected by the brain–gut axis, which controls intestinal peristalsis [15,16]. Soluble dietary fiber was administered to a constipation population to evaluate its effect, and was found to efficiently relieve constipation [11], indicating its remarkable application prospects in relieving constipation. When studying the effect and mechanism of soluble dietary fiber from corn husks on loperamide-induced constipation in mice, Zeng Xiangrui et al. confirmed that soluble dietary fiber from corn husks can alleviate constipation by regulating neurotransmitter levels and reshaping gut microbiota and metabolite levels. In addition, when combined with probiotics, it can also alleviate constipation-induced colitis [17]. When studying the effect and mechanism of compound dietary fiber on constipation in mice, Lv Quanhong et al. found that compound dietary fiber can alleviate inflammation and defecation by regulating the composition of intestinal microbiota and promoting the secretion of SCFAs [18]. Hawthorn belongs to the Rosaceae family and is a plant of the Rosaceae genus. It is a medicinal and edible resource with various pharmacological effects such as lowering blood lipids, anti-aging, anti-cancer, and anti-fatigue [19,20]. Hawthorn residue is the solid residue left after deep processing of hawthorn in factories, and its utilization rate is generally very low. Many dietary fibers in hawthorn residue have not been fully utilized. Studies on the efficacy of soluble dietary fiber from hawthorn (HSDF) in relieving constipation are insufficient. In this study, loperamide hydrochloride was injected into the stomach for modeling. Then the function and mechanism of HSDF in relieving constipation were comprehensively analyzed based on multiple aspects such as hormones, metabolism, intestinal flora, and water ion protein, to provide a theoretical basis for the development of a hawthorn pomace dietary fiber functional food.

## 2. Materials and Methods

### 2.1. Materials and Reagents

The red hawthorn with swallow flesh was obtained from Kuancheng Manchu Autonomous County, Chengde, China (latitude: 40.611391, longitude: 118.485313). It was picked in October 2020 at a maturity of 8 points. The selected hawthorn was moderately soft and hard, with a bright color and a slightly sweet taste.

Male Kunming (KM) 3-week-old mice were obtained from Beijing Vital River laboratory animal technology Co., Ltd., Beijing, China; loperamide hydrochloride from Xian Janssen Pharmaceutical Ltd., Xi’an, China; maren soft capsules from the Central Pharmaceutical Co., Tianjin, China; ELISA kits NO(AKNM005M), MDA(AKFA013M), MTL(EKMO25001), GAS(EKMO25002), VIP(EKMO1033), SP(EKMO29001), and SS(EKMO30011) from Boxbio, Beijing, China; BCA protein concentration determination kit (P0010) from Beyotime Biotechnology, China, Shanghai, China; mouse McAb β-Action (66009-1 Ig) from Proteintech Group, Inc., Wuhan, China; rabbit polyclonal antibody AQP3 (BS3671), rabbit polyclonal antibody AQP8 (BS71,279), rabbit polyclonal antibody AQP9 (BS71,280), rabbit polyclonal antibody IL-1β (A00101-1), rabbit polyclonal antibody IL-6 (BA4339-2), and rabbit polyclonal antibody TNF-α (A00002-2) from NovaTeinbio, Inc., Boston, MA, USA; HRP-labeled sheep anti-mouse secondary antibody (BA1051) and HRP-labeled sheep anti-rabbit secondary antibody (BA1054) from Wuhan Boster Biological Technology, Ltd, Wuhan, China; acetic, propionic, butyric, isobutyric, valeric, isovaleric, caproic, and isohexanoic acids (standard) from sigma, Shanghai, China; and α-amylase (100,000 U/g), glucoamylase (100,000 U/g), neutral protease (100,000 U/g), glucosidase (200 U/g), and cellulase (10,000 U/g) from Shanghai yuanye Bio Technology Co., Ltd., Shanghai, China.

### 2.2. Preparation of Hawthorn Pomace Soluble Dietary Fiber

The hawthorn fruits were denucleated, and a beating machine was used to beat them; they were then filtered to obtain hawthorn residue. The hawthorn residue was cut in a 45 °C water bath for 2 h at a 1:3 ratio of hawthorn fruit pulp and acidified ethanol (75% ethanol/25% 1% HCl), and the wet fruit residue was obtained via standing and suction filtration. The wet fruit residue was dried at 45 °C for 8 h (drying oven DHG-9073A, Shanghai instrument equipment Co., Ltd., Shanghai, China), crushed using a grinder (FSD-70, Shanghai Jiading grain and editable oil instrument Co., Ltd., Shanghai, China), and sifted through 60 meshes to obtain the dry hawthorn fruit residue. Then 1 g of dried hawthorn pomace was added to distilled water at a ratio of 1:10 (g/mL), microwave-treated at 480 W for 3 min, and cooled to 45 °C after standing. Afterwards, complex enzymes (α-amylase 0.1%, cellulase 0.5%, neutral protease 0.6%) were added and the mixture was hydrolyzed at a constant temperature of 45 °C and pH 5.0 for 120 min. Then, the samples were boiled at 95 °C for 10 min, followed by suction filtration to obtain the supernatant, with addition of anhydrous ethanol to the supernatant at a volume ratio of 1:4. After standing for 12 h for ethanol precipitation, and 3500× *g* centrifugation (TDZ5-WS, Changsha Xiang Yi Centrifuge Instrument Co., Ltd., Changsha, China) for 15 min to obtain the precipitate sequentially, the precipitate was freeze-dried (LGJ. 30D, Beijing SI HUAN Scientific Instrument Factory Co., Ltd., Beijing, China) to obtain HSDF, at an extraction rate of 70% and purity of 82.3%.

### 2.3. Structural Characterization of Hawthorn Pomace Soluble Dietary Fiber

#### 2.3.1. Fourier Transform Infrared Spectroscopy

The HSDF sample and KBr were ground into a powder of less than 1 μm in an agate mortar at a ratio of 1:100 (*w*/*w*). After processing with a vacuum tablet press, the sample was placed into the optical path for infrared spectral scanning (Nicolet 5700, Thermo Nicolet, MA, USA) within a scanning range of 4000–400 cm^−1^.

#### 2.3.2. Observation of Hawthorn Pomace Soluble Dietary Fiber

The HSDF sample was analyzed using the scanning electron microscope (SEM), and gold plating was conducted on the sample using ion spraying (E-1010, Hitachi Limited, Tokyo, Japan) for 80 s. The micrograph was digitally recorded using a scanning electron microscope (SU-8010, Hitachi Limited, Tokyo, Japan) with a magnification of 5000 times.

#### 2.3.3. X-ray Diffraction Analysis

Using a working voltage of 30 KV and a working current of 20 mA, a diffractometer (D8 Advance, Bruker, Beijing, China) with a diffraction angle ranging from 2–80° and a scanning speed of 2 °/min was used to obtain the XRD spectrum of HSDF.

#### 2.3.4. Determination of Hawthorn Pomace Soluble Dietary Fiber Molecular Weight

Based on the method report by Song et al. with slight modification, the molecular weight was estimated by gel permeation chromatography (GPC) analysis [21], and the standard was polyethylene glycol (PEG). First, the sample was dissolved in water to prepare a solution with a concentration of 5 mg/mL. The mobile phase was set to 0.1 M sodium nitrate solution, the flow rate 1 mg/mL, the column box temperature 40 °C, and the detector temperature 40 °C. A Waters Ultrahydrogel gel permeation chromatographic column, 7.8 × 300 mm, and a 2414 differential refractometer detector (waters 1515, waters, MA, USA) were used.

#### 2.3.5. Composition of Monosaccharide

HSDF monosaccharide analysis was performed using high-performance anion exchange chromatography. The mass of different monosaccharides was determined according to the absolute quantitative method, and the molar ratio calculated according to the molar mass of monosaccharides. First, 5 mg of the sample was accurately weighed and placed in an ampoule bottle. Then, 2 mL of 3 M TFA was added and the mixture was hydrolyzed at 120 °C for 3 h. The acid hydrolysis solution was accurately absorbed, transferred to the pipe, and blow-dried with nitrogen. Then 5 mL water was added for vortex mixing, 50 μL was absorbed, and 950 μL of deionized water was added. This was followed by centrifugation at 12,000 rpm for 5 min. The cleaning IC was taken for analysis. The chromatographic column was a DionexCarbopacTMPA20 (3 × 150 mm); mobile phase A: H_2_O; mobile phase B: 15 mM NaOHC, 15 Mm NaOH, and 100 mM NaOAC; flow rate: 0.3 mL/min; injection volume: 5 µL; column temperature: 30 °C; detector: electrochemical detector.

### 2.4. Animal Treatment

All animal experiments were approved by the Animal Ethics Committee of Hebei Normal University of Science and Technology (No. 2001). Forty-five healthy male KM mice (SPF grade 31 ± 2 g), adapted to feeding for 7 d, were randomly divided into 5 groups; 9 mice in each group. They were divided into blank control group C, loperamide hydrochloride model group M (10 mg/kg bw), maren soft capsule (HS) positive control group HS (0.4 g/kg bw), low-dose group LHDF (1.0 g/kg bw), and high-dose group HHDF (2.0 g/kg bw). In addition to group C, other groups received 10 mg/kg bw of loperamide hydrochloride every day at 7:00 a.m., and group C received the same quality of distilled water. At 9:00 a.m., the mice in the HS, LHDF, and HHSD groups were gavaged with corresponding quality test samples. KM mice received unrestricted access to drinking water, had their bedding changed twice a week, were kept pathogen free under a light cycle (12/12 h), and were raised at (22 ± 2) °C and (50 ± 10)% relative humidity [22]. The mental state, activity, hair glossiness, appetite, and defecation of the mice were observed every day. The mice were weighed every three days and the dosage adjusted according to their weight. The defecation experiment was conducted after 45 consecutive days of sample administration. For small intestine propulsion and index detection, the same batch of KM mice continued to receive samples by gavage for 7 days after the defecation experiment.

### 2.5. Evaluation of Defecation Function of Constipation Mice

After the final sample administration, the mice in each group were fed with water as usual during the 24 h fasting period. The mice in groups M, HS, LHDF and HHDF were given loperamide hydrochloride (10 mg/kg bw) by gavage, and the mice in group C were given distilled water by gavage; 30 min after oral administration of loperamide hydrochloride, 0.2 mL of ink paste (5% ink + 10% gum arabic) was administered to each dose group. Timing was started after gavage, and food and drink were administered normally. The time it took for mice to discharge the first black stool, and the number and quality of black stools within 6 h were recorded. After the defecation experiment, mice were given corresponding samples by gavage according to the animal treatment procedure discussed in Section 2.4 after 24 h of normal diet. Within 24 h, after 7 d of normal gavage, mice in each group stopped eating but did not stop drinking water. After 25 min, eyeball blood was taken and the serum was immediately centrifuged to determine the intestinal hormone. The mice were killed via blood decapitation, and the abdominal cavity was opened on the sterile operating platform. The intestinal segments from the upper end to the pylorus and from the lower end to the ileocecal region were cut, placed on the tray sprinkled with normal saline, and gently pulled into a straight line. After natural retraction, the length of the intestinal segment was regarded as the “total length of the small intestine”, and the length from the pylorus to the ink front was the “ink advance length”.

Fecal particles were collected near the colon approximately 8 cm away from the anus and placed in a sterile EP tube to determine the intestinal flora and short-chain fatty acids. Then, the colon was cut into two sections; one section was stored at −80 °C for testing, and the other was fixed in paraformaldehyde (4% PFA) solution for standby.

The ink propulsion rate (%) = ink propulsion length (cm)/total length of small intestine (cm) × 100.

### 2.6. Histopathology

The fixed colon sample was stained with H&E and AB–PAS, dehydrated using alcohol, and made transparent using xylene. The transparent tissue block was immersed in melted paraffin and completely embedded. It was fixed on a microtome (RM 2016 rotary microtome, Leica Microsystems AG, Wetzlar, Germany) and cut into 4 μM thick sheets. The corresponding staining agents—hematoxylin eosin or alcian blue, periodate, and Schiff’s reagent—were used for staining and observation.

### 2.7. Physiological Index Measurement

First, 1.0 g of colon was accurately weighed, dispersed in a 2.0 mL centrifuge tube, and centrifuged at 11,300× *g* at 4 °C (VELOCITY 18R Pro desktop multi-function centrifuge, Shanghai, China) for 10 min. The supernatant was obtained, and the motilin (MTL), gastrin (GAS), vasoactive intestinal peptide (VIP), substance P (SP), and somatostatin (SS) contents in the colon were determined with reference to the instructions of the ELISA kit. The NO and MDA contents in serum were directly determined using the ELISA kit.

### 2.8. Western Blot Analysis

The appropriate amount of cut colon tissue was placed into an EP tube and 200 μL LRIPA pyrolysis liquid homogenate was added; the homogenate was then placed on ice for 30 min, fully decomposed, and then the supernatant was centrifuged at 13,300× *g* and 4 °C. The BCA protein concentration determination kit was used to determine the protein concentration, and the OD value was measured using a microplate reader (ReadMax1900, Shanghai Sandstrop Biotechnology Co., Ltd., Shanghai, China) at a wavelength of 562 nm; the loading volume was 40 μg. After electrophoresis, SDS-PAGE glue was stripped for semi-dry membrane transfer (PVDF membrane, 100 V, 1.5 h). After membrane conversion, the membrane was sealed with 5.0% skimmed milk for 1 h; we then incubated β-actin, IL-1β, IL-6, TNF-α, AQP3, AQP8, and AQP9 primary antibodies (1:5000) for 2 h. The PVDF membrane was transferred into the membrane washing box containing phosphate buffer solution, and washed 3 times (10 min/time) following the addition of the proper amount of TBST. The HRP-labeled secondary antibody (1:10,000) was incubated for 2 h, and transferred to the membrane washing box containing phosphate buffer solution for washing 3 times (10 min/time). The β-actin is an internal parameter.

### 2.9. Determination of Short-Chain Fatty Acids in Colon Contents

An appropriate amount of the sample was placed into a 2 mL centrifuge tube, and 50 μL 15% phosphoric acid plus 125 μg/mL internal standard (methylvaleric acid) solution, 100 μL ether, and 400 μL homogenate were added for 1 min. The mixture was centrifuged at 4 °C 13,300× *g* for 10 min, and the supernatant was obtained for testing. Detection conditions of the GC-MS on the upper machine were: chromatographic column Agilent HP-INNOWAX capillary column (30 m × 0.25 mm ID × 0.25 μm); split injection; injection volume 1 μL; split ratio 10:1. The sample inlet temperature was 250 °C, the ion source temperature 300 °C, and the transmission line temperature 250 °C. The temperature programming was commenced at 90 °C, then raised to 120 °C at 10 °C /min, and to 150 °C at 5 °C/min. Finally, the temperature was raised to 250 °C at 25 °C /min for 2 min. The carrier gas was helium, and the carrier gas flow rate was 1.0 mL/min. Mass spectrometry (ISQ LT, Thermo Fisher Scientific, Shanghai, China): electron bombardment ionization (EI) source, SIM scanning mode, electron energy 70 eV.

### 2.10. High Throughput Sequencing of 16S rRNA of Mouse Intestinal Flora

The extracted DNA was detected using an Equalbit dsDNA HS detection kit. The highly variable region of 16S rRNA was amplified using a PCR primer, and the next-generation sequencing was performed using 20–30 ng DNA as a template. The concentration of the DNA library was detected using a microplate reader, and double-ended sequencing was performed on the Illumina MiSeq platform. In this study, 16S rRNA amplification and sequencing were completed by Shanghai Biotech Co., Ltd., Shanghai, China.

### 2.11. Statistical Analysis

SPSS19.0 statistical software was used for statistics, and the data were represented by “X ± SD”. One-way variance (ANOVA) was used for statistical analysis, and Tukey’s comparison test was used to obtain a significant difference (*p* < 0.05 was significant, *p* < 0.01 was extremely significant, and *p* < 0.001 was highly significant). Data statistics and analysis were performed and GraphPad Prism software (Version8.0, GraphPad software, Inc., San Diego, CA, USA) was used to draw images.

## 3. Results

### 3.1. Hawthorn Pomace Soluble Dietary Fiber Structure and Composition Analysis

**Figure 1:**
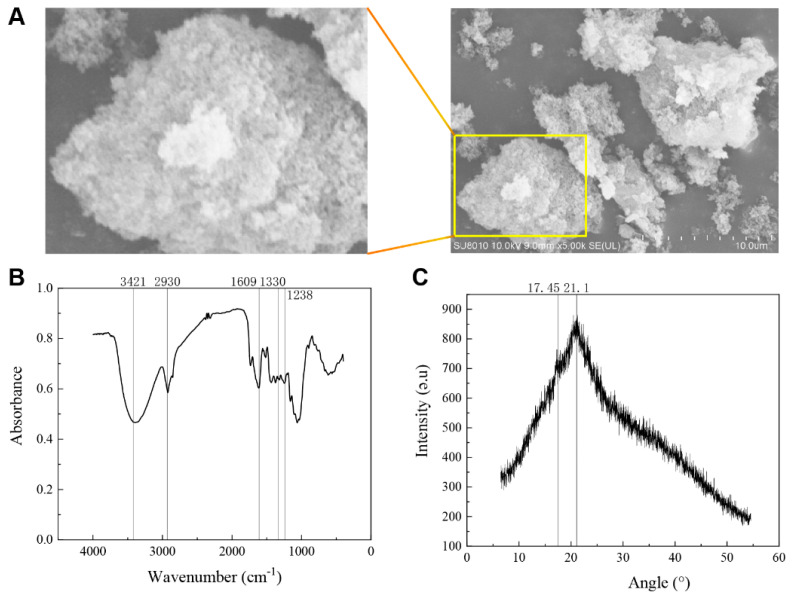
Structural characteristics of hawthorn soluble dietary fiber. (**A**) Hawthorn pomace soluble dietary fiber (HSDF) Scanning Electron Microscopy (Hitachi Ltd., Tokyo, Japan) (SEM 5000×); (**B**) HSDF Fourier transform infrared spectrogram; (**C**) X-ray diffraction pattern.

As shown in Figure 1A, the surface of HSDF is uneven and loose, and a fault structure is formed. The numerous small particles on the surface resemble snowflakes and increase the specific surface area. Their pore sizes are conducive to the adsorption of more water and oil molecules by SDF, and improve the water-holding capacity and expansion rate of HSDF [23].

Figure 1B shows the Fourier transform infrared spectrum of HSDF. Among them is the hydroxyl stretching vibration peak of 3421 cm^−1^, the C-H stretching vibration peak of carbohydrate methyl of 2970 cm^−1^, the C=O (CHO-) stretching vibration peak of 1635 cm^−1^, the C=O (-COO) and C-O (-COO) stretching vibration peaks of 1330 cm^−1^ and 1242 cm^−1^, respectively, the O-H bending vibration peak of 1049 cm^−1^, and the ester group peak of 1744 cm^−1^. These absorption peaks prove that HSDF has the characteristic structure of polysaccharides. The molecule contains an ester group.

The HSDF molecular weight results (Table 1) show that the sample has two kinds of molecular weight components. The low polydispersity index indicates that the molecular distribution is relatively dense, where the higher and lower average molecular weights are 17,973 Da and 3381 Da, respectively. Moreover, the lower molecular weight accounts for 29% of the total weight, while the weight-average molecular weight of most soluble dietary fibers is more than 1 × 10^5^ Da [24], which indicates that microwave enzymolysis-treated SDF belongs to the medium and low molecular weight dietary fibers. The lower dose is more easily absorbed by the human body, exhibits a higher anti-inflammatory effect, and can increase the level of beneficial bacteria in the colon of mice [25].

HSDF contains nine kinds of monosaccharides: fucose, rhamnose, arabinose, galactose, glucose, xylose, mannose, galacturonic acid, and glucuronic acid (Table 2). Galacturonic acid is the main monosaccharide in HSDF. Galacturonic acid is a polymer linearly linked by α-1,4 glycosidic bonds and is the main monosaccharide in pectin. The abundance of galacturonic acid indicated that pectin might be an important component of HSDF. Arabinose, galactose, glucose, and xylose are present in HSDF, indicating that hemicellulose polymer is one of the main components of hawthorn fruit residue SDF. A small amount of fucose was detected in HSDF, and the existence of fucose and galacturonic acid made HSDF reducible and relatively stable.

The above results show that the structure of HSDF corresponds to the crystal structure of natural cellulose type I, the characteristic functional groups of sugars, and that galacturonic acid is the main monosaccharide component. The HSDF structure is not compact; it is snowflake-shaped with low molecular weight, which is more conducive to water retention, adsorption, and oxidation resistance of dietary fiber.

### 3.2. Hawthorn Pomace Soluble Dietary Fiber Improves the Physiological Condition of Mice

**Figure 2:**
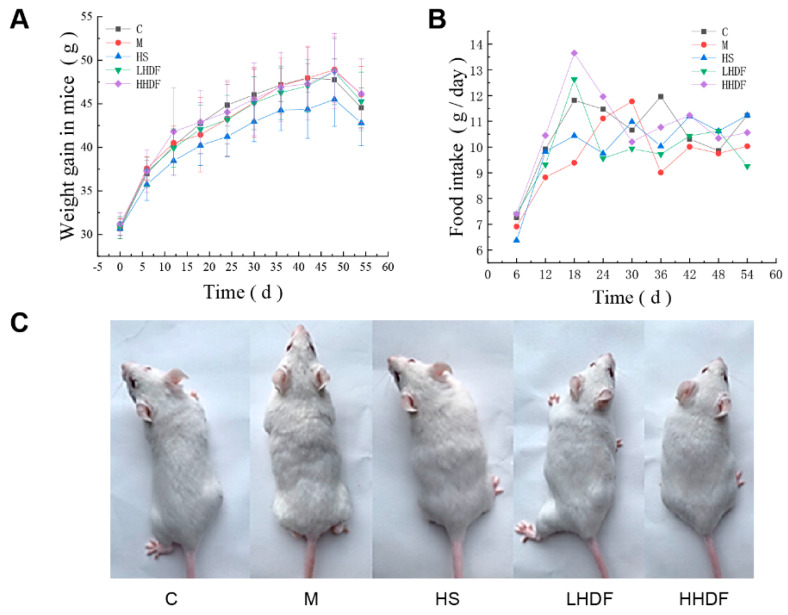
Effects of HSDF on physiological indexes of mice. (**A**) The body weight was recorded every three days and the amount of food given was adjusted based on body weight for each group of mice. (**B**) The remaining food was weighed every three days, and food to the specified weight was added according to the weight of the remaining food: 150 g of food per group for 1–6 days, 180 g of food per group for 6–12 days, 210 g of food per group for 12–18 days, and 250 g of food per group for 18–54 days. (**C**) On the last day, before the start of the gastric ink in the experimental mice, the hair status of each group of mice was observed: C, blank control group; M, loperamide hydrochloride model group; HS, maren soft capsule (0.4 g/kg bw); LHDF, low-dose HSDF (1.0 g/kg bw); HHDF, high-dose HSDF (2.0 g/kg bw). Results are expressed as mean ± SD (*n* = 9).

The feeding, drinking, and activity of the five groups of mice were normal, without vomiting and abnormal death, indicating that HSDF and loperamide hydrochloride at the dose and time used in the experiment had no effect on the normal physiological conditions of mice. Figure 2A shows that there was no difference in the initial weight of mice in each group. In the subsequent growth process, the weight growth rate of mice in the HS treatment group was slightly lower than that of mice in other groups. There are no significant differences among the C, M, LHDF, and HHDF groups (*p* > 0.05), indicating that HSDF had no adverse effect on the weight change of mice during the consumption period. The mice in the HS group may have been slightly lighter in weight than other groups owing to the side effect of constipation drugs on gastrointestinal stimulation. As seen in Figure 2C, the coat color of the HS group was dull, indicating the unsuitability of certain constipation relief drugs for the long-term treatment of constipation. As shown in Figure 2B, the food intake of the HS and sample groups was not significantly different from that of group C, indicating that HSDF and loperamide hydrochloride did not affect the normal physiological activities of mice. Figure 2C shows that the fur was more shiny in groups C and HHDF than that in groups HS and M. In group M, the hair was uneven and incomplete, and the fur color was relatively dark, suggesting that constipation affects the luster of hair and skin, and HHDF can significantly improve this phenomenon.

### 3.3. Hawthorn Pomace Soluble Dietary Fiber Improves Defecation Indexes of Mice

**Figure 3:**
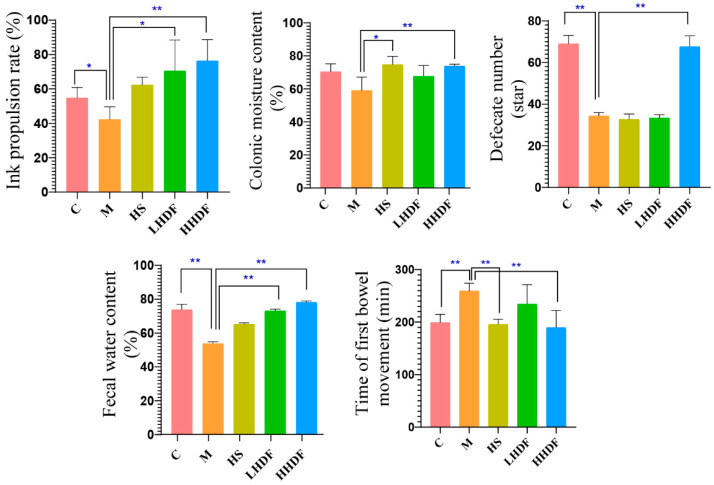
Pathological analysis of colon tissue in each group of mice treated with HSDF. C: blank control group; M: loperamide hydrochloride model group; HS: maren soft capsule (0.4 g/kg bw); LHDF: low-dose HSDF (1.0 g/kg bw); HHDF: high-dose HSDF (2.0 g/kg bw). Results are expressed as mean ± SD (*n* = 5). ** *p* < 001, * *p* < 0.05.

Compared with group C (Figure 3), the time of first bowel movement in group M was significantly prolonged, the ink propulsion rate was reduced, the fecal water content and colonic moisture content were significantly reduced (*p* < 0.01 or *p* < 0.05), and the defecate number was significantly reduced within 6 h (*p* < 0.01), indicating that the model was successful. There was no significant difference between HS and M in terms of ink advance rate, defecation quantity, and fecal water content, but there were significant (*p* < 0.05) and extremely significant (*p* < 0.01) differences in colon water content and first bowel movement time. The fecal water content, ink penetration rate, colon water content, and bowel movements of mice in the low-dose and high-dose HSDF groups were significantly higher than those in the M group—particularly HHDF (*p* < 0.01)—at 45.65%, 89.90%, 25.11%, and 97.06%, respectively. There was no significant difference in the first bowel movement time between the M group and the LHDF group, but the bowel movement time was significantly longer than for other drug groups and the C group (*p* < 0.01), and that of the HHDF group was 65.5 min less than that of group M. The results suggested that low- and high-dose HSDF could effectively promote intestinal peristalsis and relieve constipation symptoms in mice with constipation, and the effect in the high-dose group was stronger than that in the low-dose group.

### 3.4. Hawthorn Pomace Soluble Dietary Fiber Improves Gastrointestinal Hormone Levels in Mice with Constipation

HSDF significantly improved the abnormal gastrointestinal hormone levels in KM constipation mice caused by loperamide hydrochloride, as shown in Table 3. Compared with group C, the levels of MTL, GAS, SP, and VIP in group M were significantly lower (*p* < 0.01), while the levels of SS were significantly higher (*p* < 0.01). Compared with group M, the contents of MTL, GAS, SP, SS and VIP in the LHDF and HHDF groups were significantly increased (*p* < 0.01 or *p* < 0.05). The MTL, GAS, SP, and VIP contents in the HHDF group increased by 64.65%, 40.19%, 48.94%, and 133.36%, respectively (HHDF group vs. M group), while those in the LHDF group increased by 39.03%, 21.03%, and 41.23% (LHDF group vs. M group). SS content in the HHDF and HS groups was significantly lower than that in M group (*p* < 0.01), and decreased by 23.30% in the HHDF group (HHDF group vs. M group). The MTL, GAS, SP, and VIP content in the HHDF group were 18.43%, 15.83%, 5.46%, and 65.03% higher than those in the LHDF group. The content of inhibitory intestinal hormone SS in the HHDF group was 90.93% lower than that in the LHDF group.

To investigate the effect of HSDF on the oxidative stress of mice with constipation, the NO and MDA contents in the serum of mice with constipation were determined. Compared with group C, the NO and MDA content in group M was significantly higher (*p* < 0.01), while the NO and MDA content in the HHDF group was significantly lower than that in group M by 46.77% and 96.67%, respectively. Moreover, the content in the HHDF group was lower than that in the LHDF group, indicating that HSDF could reduce the content of inhibitory gastrointestinal hormones, thereby promoting colon peristalsis and accelerating defecation.

### 3.5. Hawthorn Pomace Soluble Dietary Fiber Reduces Inflammation of Colon Tissue

**Figure 4:**
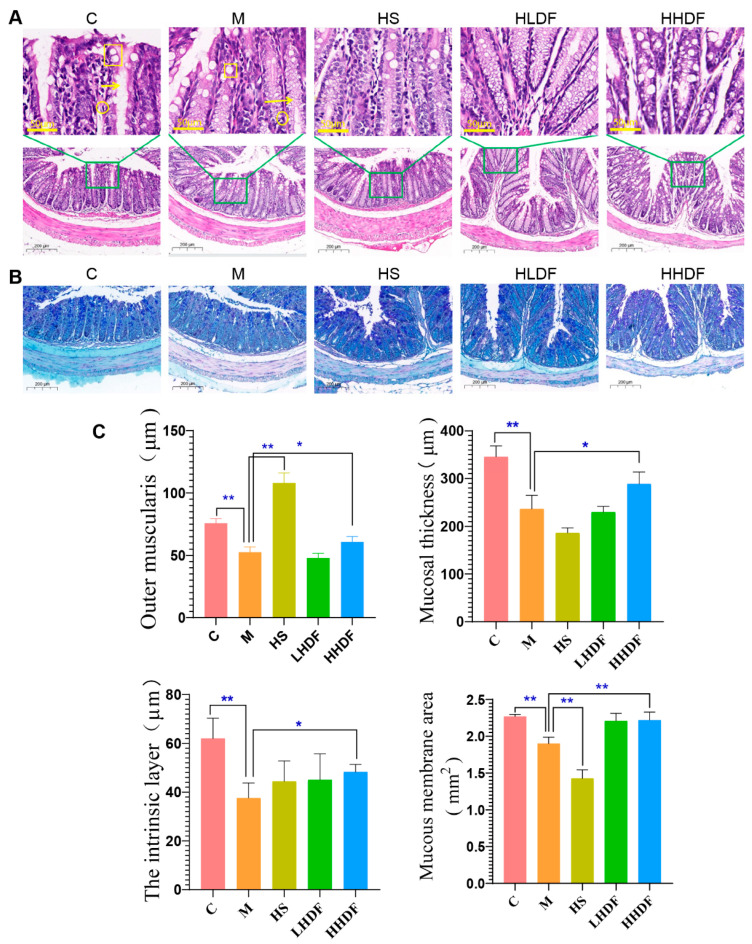
Pathological observation of colonic inflammatory constipation. (**A**) Hematoxylin and eosin (H&E) staining of mouse colon. (**B**) AB-PAS staining of mouse colon. (**C**) Mouse histopathological data statistics. The scale is 50 μm and 200 μm. H&E and AB-PAS. From top to bottom: Round—inflammatory cells; Square—goblet cells; Arrow—recess; C—blank control group; M—loperamide hydrochloride model group; HS—maren soft capsule (0.4 g/kg bw); LHDF—low-dose HSDF (1.0 g/kg bw); HHDF—high-dose HSDF (2.0 g/kg bw). Results are expressed as mean ± SD (*n* = 3). ** *p* < 0.01, * *p* < 0.05.

The thickness of colonic mucus and the decrease in goblet cell atrophy may cause constipation to a certain extent [26]. The H&E staining in Figure 4A,C show that, compared with group C, the nuclei of colon tissue in M group mice undergo pyknosis, with more inflammatory cell infiltration and reduced mucosal crypt deformation. Moreover, there were significant differences (*p* < 0.01) between group M and group C in terms of outer muscle layer, mucosal thickness, inner muscle layer, and mucosal area, indicating that after drug induction, the mucosal thickness of the colon lumen and colon mucosa in group M animals shrank. Compared with the M group, the HS, LHDF, and HHDF groups showed a decrease in inflammatory cells in colon tissue and a significant increase in crypts, and the HHDF group of mice showed the best improvement effect, significantly increasing the thickness and area of the mucosa, as well as the thickness of the inner and outer muscle layers. The results showed that HSDF could attenuate the pathological changes in the gastrointestinal tract and reduce inflammation in mice with constipation induced by loperamide hydrochloride. The AB-PAS staining diagram in Figure 4B,C show that compared with Group C, the mucosal goblet cells stained with AB-PAS in the colon tissue of Group M atrophy and mucus decrease significantly. Compared with group M, the secretion of goblet cells and mucus in the intervention group recovered to a certain extent compared with that in the model group. The recovery of goblet cells and mucosa in the HSDF intervention group indicated that HSDF could relieve constipation by repairing the colon tissue.

**Figure 5:**
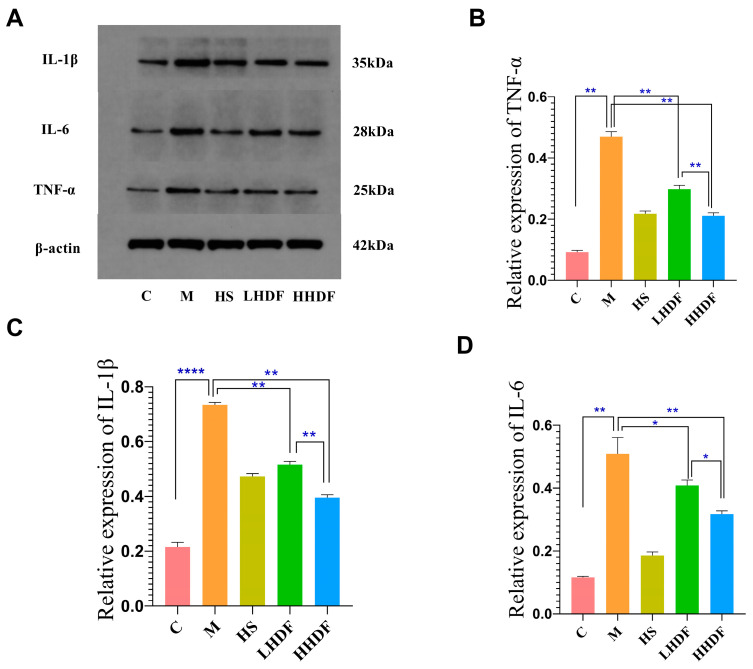
The effect of HSDF on the expression of inflammatory factor proteins in various groups of mice. (**A**) WB results of the expressions of inflammatory factors IL-6, IL-1β, and TNF-α. (**B**) Relative expression of TNF-α in each group. (**C**) Relative expression of IL-1β in each group. (**D**) Relative expression of IL-6 in each group. C: blank control group; M: loperamide hydrochloride model group; HS: maren soft capsule (0.4 g/kg bw); LHDF: low-dose HSDF (1.0 g/kg bw); HHDF: high-dose HSDF (2.0 g/kg bw). Results are expressed as mean ± SD (*n* = 3) **** *p* < 0.0001, ** *p* < 0.01, * *p* < 0.05.

To further investigate the anti-inflammatory mechanism of HSDF in constipation, the protein expressions of inflammatory factors IL-6, IL-1β, and TNF-α were detected (Figure 5). Compared with group C, the relative expressions of IL-6, IL-1β, and TNFα in group M were all relatively high (*p* < 0.01). Compared with group M, the relative expression levels of three inflammatory factors in the HHDF of the HS group and LHDF groups were significantly reduced: IL-6 was decreased by 63.46%, 19.84%, and 37.72%; IL-1β by 35.56%, 29.70%, and 46.19%; and TNF-α by 53.62%, 36.60%, and 55.11%, respectively (*p* < 0.01). Compared with the HHDF group, the protein expression levels of IL-6, IL-1 β, and TNF-α were lower in the HHDF group, indicating that higher concentrations of HHDF have a better inhibitory effect on the expression of inflammatory factors. The results were consistent with the section staining results, indicating that HSDF could repair the colon tissue, reduce intestinal inflammation, and improve the symptoms of constipation.

### 3.6. Hawthorn Pomace Soluble Dietary Fiber Regulates AQP Water Pathway Protein Expression to Relieve Constipation in Mice

**Figure 6:**
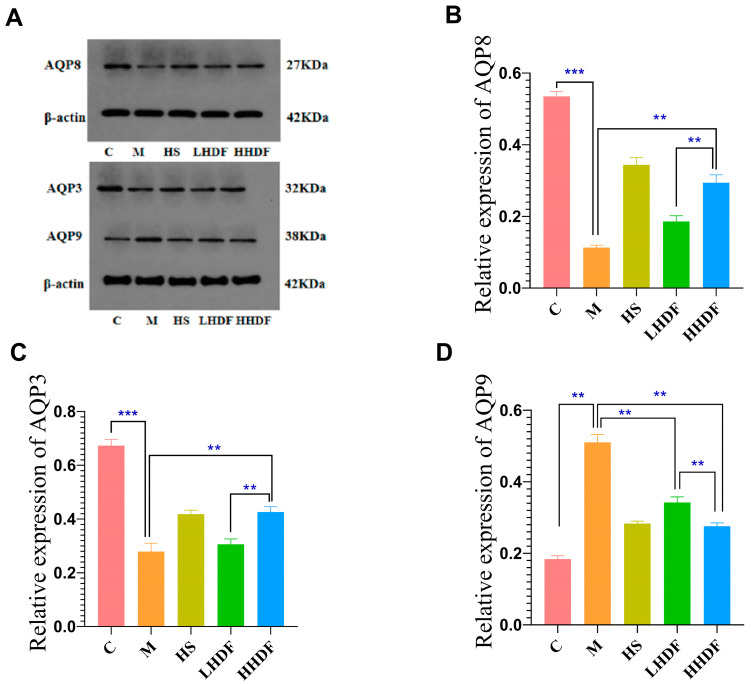
The effect of HSDF on the expression of aquaporin in various groups of mice. (**A**) WB results of the expressions of AQP8, AQP3 and AQP9. (**B**) Relative expression of AQP8 in each group. (**C**) Relative expression of AQP3 in each group. (**D**) Relative expression of AQP9 in each group. C: blank control group; M: loperamide hydrochloride model group; HS: maren soft capsule (0.4 g/kg bw); LHDF: low-dose HSDF (1.0 g/kg bw); HHDF: high-dose HSDF (2.0 g/kg bw). Results are expressed as mean ± SD (*n* = 3). *** *p* < 0.001, ** *p* < 0.01.

AQPs play an important role in colonic water metabolism. Abnormal AQP expression can lead to intestinal water balance disorder. The levels of AQP3 and AQP8 in colonic tissues of Group M were significantly lower than those in colonic tissues of Group C (*p* < 0.01) (Figure 6). However, the levels of AQP3 and AQP8 in the colon tissue of the LHDF and HHDF groups were higher than those in the M group colon tissue, but lower than those in the C group colon tissue (*p* < 0.01), indicating that HDF can alleviate inflammation by inhibiting the levels of AQP3 and AQP8 proteins. Compared with the HHDF group, the LHDF group had higher levels of AQP3 and AQP8 in the colon tissue, and there was a highly significant difference between the two groups (*p* < 0.01). Compared with group M, the levels of AQP3 and AQP8 in LHDF were not significantly different. Compared with group M, the AQP3 and AQP8 levels in the colonic tissues of HHDF group were significantly higher than those of group M (*p* < 0.01), with values of 52.67% and 164.54%, respectively. AQP9 belongs to inhibitory aquaporin, and the level in the HHDF group was 45.88% lower than that in the M group (*p* < 0.01). These results indicate that HSDF can regulate aquaporins and constipation symptoms.

### 3.7. Hawthorn Pomace Soluble Dietary Fiber Improves the Composition of Short-Chain Fatty Acids in the Colon Contents of Mice with Constipation

**Figure 7:**
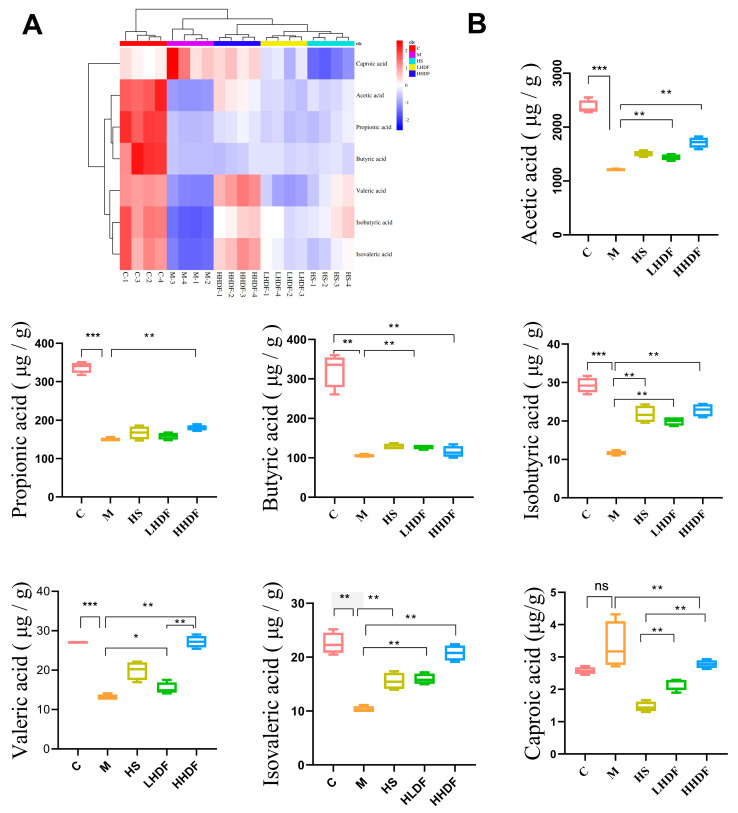
The effect of HSDF on the content of short-chain fatty acids in each group of mice. (**A**) Heat map of SCFAs. Red and blue indicate high and low levels, respectively. (**B**) Effect of HSDF on SCFA content in colon of mice. C: blank control group; M: loperamide hydrochloride model group; HS: maren soft capsule (0.4 g/kg bw); LHDF: low-dose HSDF (1.0 g/kg bw); HHDF: high-dose HSDF (2.0 g/kg bw). Results are expressed as mean ± SD (*n* = 4). *** *p* < 0.001 ** *p* < 0.01, * *p* < 0.05.

Dietary fiber can ferment into short-chain fatty acids (SCFAs) and other substances from intestinal microorganisms under anaerobic conditions in the large intestine, which is closely related to the composition and content of the intestinal flora. As seen in the heat map of short-chain fatty acids in the colon contents in Figure 7A, loperamide hydrochloride causes abnormal SCFAs, and the dietary supplement of HSDF can reverse this phenomenon, particularly HHDF, which is close to the normal group. Interestingly, HHDF is more effective than the positive control HS at the experimental dose. Further analysis of SCFA content in Figure 7B shows that compared with group C, the mice in group M had reduced content of six SCFAs in the colon, including acetic, protonic, isobutyric, butyric, isovaleric, and valeric acids, and increased production of caproic acid. This may be attributed to constipation causing disorder in the intestinal flora and changing microbial metabolism. At the same time, the decrease in SCFA content affects the development of intestinal epithelial cells, is not conducive to the repair of intestinal mucosa, affects intestinal movement, and adversely impacts the host immune system. Our results showed that HSDF restored the SCFA content in the colon of mice. Compared with group M, LHDF and HHDF significantly increased acetic acid, propionic acid, isobutyric acid, isovaleric acid, and valeric acid (*p* < 0.05)—especially in the HHDF group—by 41.91%, 19.85%, 96.76%, 101.63%, and 108.44%, respectively; However, in terms of butyric acid, there was not much difference in content between the LHDF and HHDF groups and the M group, indicating that HDF had a relatively small impact on the butyric acid content in the intestine. Compared with the M group, the HS group significantly increased the content of acetic acid, butyric acid, isobutyric acid, valeric acid, and isovaleric acid, while reducing the content of hexanoic acid, with little effect on propionic acid; Compared with the HLDF group, the HS group had an increased content of hexanoic acid, but there was not much difference between the two groups in terms of other short-chain fatty acids, indicating that the impact of HLDF on short-chain fatty acids is relatively small. Compared with the HHDF group, the HS group had a significantly increased content of acetic acid, hexanoic acid, valeric acid, and isovaleric acid, indicating that HHDF has a greater impact on the content of short-chain fatty acids in the intestine.

### 3.8. Hawthorn Pomace Soluble Dietary Fiber Improves the Intestinal Flora and Relieves Constipation in Mice

**Figure 8:**
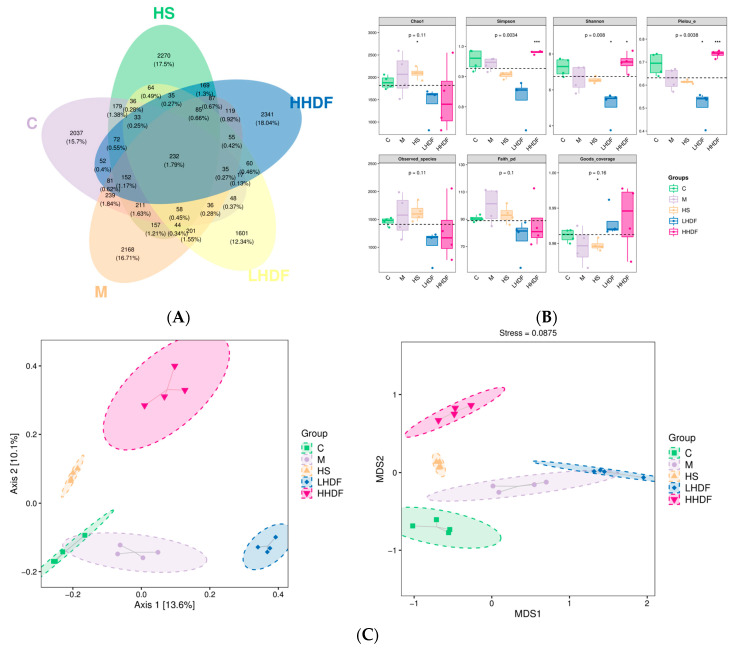
The effect of HSDF on the diversity of gut microbiota in different groups of mice. (**A**) Venn diagram. The OTUs are shared among different groups. Statistical chart of the number of microbial taxonomic units at each level. (**B**) Box plot of microbial diversity index of microbiota. (**C**) Principal coordinate analysis (PCoA). Based on weighted UniFrac distance matrix, principal components (PC) 1 and 2 explained 13.6% and 10.1% of the variance, respectively; NMDS two-dimensional sorting diagram (right). Principal coordinate analysis (PCoA) finds the principal coordinate based on the Bray–Curtis sample-to-sample distance matrix. Non-metric multidimensional scale analysis (NMDS) is similar to the PCoA analysis above, but also through the sample distance matrix dimensionality reduction decomposition, simplifying the data structure, thereby describing the distribution characteristics of the sample at a specific distance scale. C, blank control group; M, loperamide hydrochloride model group; HS, maren soft capsule (0.4 g/kg bw); LHDF, low-dose HSDF (1.0 g/kg bw); HHDF, high-dose HSDF (2.0 g/kg bw). Results are expressed as mean ± SD (*n* = 4). *** *p* < 0.001, * *p* < 0.05.

The composition and content of the intestinal flora were further determined based on SCFAs. As shown in Figure 8A, there were 2037 OTUs in group C, 2186 OTUs in group M, 2270 OUTs in group HS, 1601 OTUs in group LHDF, 2341 OTUs in group HHDF, and 232 identical OTUs in all five groups, indicating significant differences in the microbial community structure among groups. Loperamide hydrochloride and HSDF affect the intestine and change the structure of the intestinal flora.

#### 3.8.1. Expression of Alpha Diversity

Figure 8B shows that compared with Group M, the Chao1 index in the LHDF and HHDF groups was lower than that in the M and C groups, indicating that HDF can reduce the abundance of gut microbiota in mice. However, the Shannon index, Observed index, and Faith index decreased in the LHDF and HHDF groups, indicating that HSDF reduced species richness. However, the Shannon and Simpson indices of the HHDF group significantly increased, close to those of the C group, indicating that HHDF increased species diversity and was beneficial for the stability of biological systems. Compared with the M and C groups, the Pielou index in the HHDF group is relatively high, indicating its complex diversity. The coverage index of Good proves the true reliability of these data.

#### 3.8.2. Beta Cluster, Beta Diversity, and Linear Discriminant Analysis Effect Size Analysis

**Figure 9:**
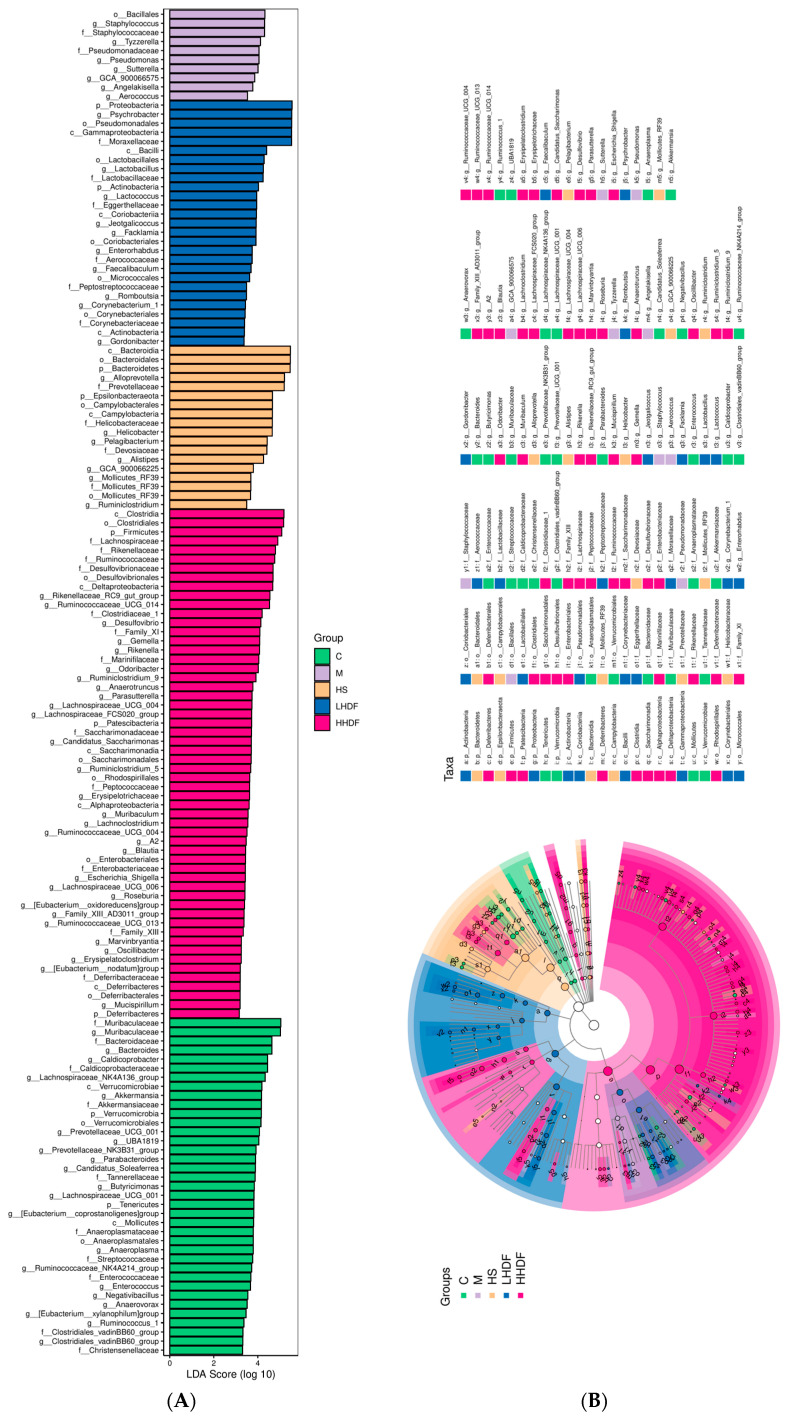
Linear discriminant analysis (LDA) histogram of microbial species diversity and evolutionary branch diagram of linear discriminant analysis effect size analysis. (**A**) A cladogram showing specific bacteria among six groups. (**B**) Linear discriminant analysis (LDA) showing scores of these specific bacteria. The LDA score indicates a significant difference caused by species abundance.

After the group alpha diversity analysis, to reflect the diversity among samples; the samples were analyzed for intergroup beta diversity. These system types played a key role in distinguishing the composition of intestinal microbiota in each group. In Figure 8C, the microbial communities in each group were similar in the PCoA and NMDS score charts. The sampling points between groups were far apart and did not overlap, indicating that dietary HSDF supplementation significantly changes the microbial composition in the intestine of mice with constipation. The LEfSe method was used to identify specific bacterial taxa. A total of 148 specific bacteria (LDA score > 3) were divided into five groups (Figure 9). There were 37 specific bacteria in group C, 10 in group M, 18 in group HS, 27 in group LHDF, and 56 in group HHDF, which showed that the differences between groups were significant, and HSDF could affect the structure of bacterial groups. Combined with the results in Figure 9, HSDF can restore the imbalance of the intestinal flora caused by loperamide hydrochloride, promote beneficial bacteria, inhibit harmful bacteria, increase bacterial diversity, and heighten intestinal stability.

#### 3.8.3. Analysis of Species Difference and Marker Species

**Figure 10:**
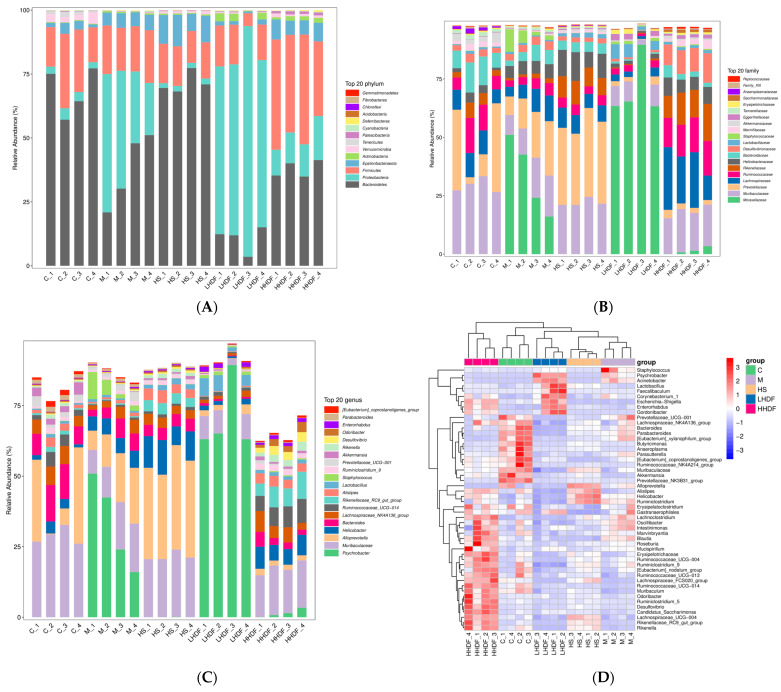
Analysis of difference of intestinal flora of mice colon by HSDF. (**A**) Histogram of the phylloscopus of species composition at the phylum level. (**B**) Histogram of phylloids composed of species at the family level. (**C**) Histogram of the composition of species at the level of the genus. (**D**) Genera-level species composition heat map of species clusters; red and blue indicate high and low levels, respectively. C, blank control group; M, loperamide hydrochloride model group; HS, maren soft capsule (0.4 g/kg bw); LHDF, low-dose HSDF (1.0 g/kg bw); HHDF, high-dose HSDF (2.0 g/kg bw).

At the Phylum level (Figure 10A), Bacteroidetes, Proteobacteria, Firmicutes, Epsilonbacteraeota, and Actinobacteria were dominant in the intestinal microbiota of each group. Bacteroidetes were the main microbiota in the intestinal microbiota of mice in groups C and HS, Proteobacteria increased in group M, and Bacteroidetes and Firmicutes decreased. Proteobacteria is the main microbiota in the intestinal microbiota of mice in the LHDF group, and the effect of improving the microbiota is not significant. Compared with group M, the HHDF group had an increased proportion of Firmicutes/Bacteroidetes and decreased Proteobacteria. The high proportion of Proteobacteria is a major causal factor of colon disease.

At the Family level (Figure 10B), the abundance of Moraxellaceae, Helicobacter, and Staphylococcus in group M was significantly higher than that in Group C, and after HHDF treatment, the abundance of Moraxellaceae and Staphylococcus decreased significantly, thus regulating the balance of the intestinal flora. In addition, Lachnospiraceae, Ruminococcaceae, Rikenellaceae, and Desulfovibrionaceae were significantly increased in the HHDF group compared with the other four groups. HHDF reduced the abundance of Prevotellaceae and Bacteroidaceae. In addition, the abundance of Helicobacteraceae was the highest in the HS group, and excessive Helicobacteraceae content increased the risk of causing colon cancer and gastric cancer, which might be one of the side effects of drug treatment.

At the Genus level (Figure 10C,D), the thermogram and histogram showed that the intestinal flora of mice at the genus level was different. There were significant differences between groups C and M among other groups. Compared with group C, the abundance of Psychrobacter, Helicobacter, and Staphylococcus in group M increased significantly, while the abundance of Muribagulaceae, Bacteroides, and Alistipes in group M decreased significantly. Compared with group M, the abundance of Psychrobacter, Muribaculateae, and Bacteroides decreased in the HHDF group. The HHDF group gained the Helicobacter and Rikenellaceae_RC9_gut_group, Alistipes, and Ruminococcaceae_UCG-014. The HHDF group recovered disorder in the intestinal flora induced by loperamide hydrochloride at the genus level to a certain extent. The abundance of Lactobacillus in the LHDF group was significantly higher than that in other groups, and that in the HHDF group exceeded that in the M group to a certain extent, indicating that HSDF was beneficial to the growth of Lactobacillus, Alistipes, and Ruminococcaceae_UCG-014, as well as the maintenance of intestinal acidic environment and health.

## 4. Discussion

Constipation is a common symptom which seriously damages patients’ health and costs a lot of medical and health care resources [27]. Chrysanthemum morifolium polysaccharide can improve constipation symptoms by promoting intestinal movement, and increasing fecal water content and intestinal hormone levels [28]. Therefore, HSDF can alleviate constipation through these pathways. We evaluated the effect and mechanism of hawthorn fruit residue dietary fiber, a natural substance, in relieving constipation. The dietary fiber extracted via the microwave-assisted enzymatic method exhibits a loose snowflake-like structure, high crystallinity, remarkable water retention and adsorption, and structural features that relieve constipation. The crystal structure of HSDF was characterized using X-ray diffraction (Figure 1C). For HSDF at 2 θ, obvious diffraction peaks appeared at 14.3°, 21.1°, 32.2°, and 40.1°, where 21.1° was the main diffraction peak, which conforms to the crystal structure of natural cellulose type I and the coexistence of the crystalline and amorphous regions. The low molecular weight of HSDF and the presence of galacturonic acid and fucose in monosaccharides give HSDF certain antioxidant activities. Previous studies have shown that the enzymatic method can increase the surface area of extracted dietary fiber, thereby exposing more water-binding sites, which is conducive to the absorption and penetration of water molecules [29] and consistent with the results of this study.

Excretion time of the first stool, intestinal propulsion rate, and fecal water content are important indicators of constipation. As shown in Figure 3, the ink advance rates of the HS group, LHDF group, and HHDF group were higher than those of the M group and C group. Compared with the M group, the ink advance rate, colon water content, and fecal water content of the HS group, LHDF group, and HHDF group were all improved; Among them, HHDF had the best improvement effect, which was 89.90%, 25.11%, and 45.65% higher than the M group, respectively; However, the HHDF group was able to better increase the number of bowel movements in mice and shorten the first bowel movement time, reducing 65.5 min compared to the M group. In summary, the HHDF group, LHDF group, and HS group all had improved bowel movement index, with HHDF having the best improvement effect. The relieving effect of HSDF on constipation caused by loperamide hydrochloride was observed.

Constipation symptoms can be alleviated by regulating gastrointestinal hormones [30]. Gastrointestinal hormones ensure the proper functioning of the digestive system through different signaling pathways [22]. MTL, GAS, SP, VIP, VIP, SS, NO, and MAD belong to the category of gastrointestinal hormones. MTL and GAS are polypeptide noncholinergic gastrointestinal hormones composed of amino acids, which can improve the contractility and tension of the gastrointestinal tract [31]. SP can affect intestinal muscle reflex, reduce inflammation, improve intestinal structure, and inhibit gastrointestinal mucosal secretion [32], and VIP can promote intestinal mucosal secretion of water and electrolytes [6]. These four hormones promote intestinal peristalsis. Gastrointestinal hormone SS can inhibit the contraction of smooth muscles, inhibit the secretion of gastrin and pepsin, and inhibits intestinal peristalsis with NO and MDA. Our results showed that HSDF increased the content of three excitatory hormones (MTL, GAS, SP) and decreased the content of three inhibitory hormones (SS, NO, MDA). Similarly, Dendrobium officinale polysaccharides increased the levels of GAS, SP, MTL, and AChE (acetylcholinesterase) in mouse serum, and decreased the levels of SS [33], which proved that HSDF had a relieving effect on constipation caused by loperamide hydrochloride, and the effect of the high-dose group was stronger than that of the low-dose group.

Low inflammation of the mucosa is a feature of functional constipation [34]. Therefore, the occurrence of constipation generally shows an increase in the content of gastrointestinal inflammatory factors and obvious damage to the gastrointestinal mucosa [35]. In the normal group, there were intact epithelial cells, the mucosa and the smooth muscle layer in the colon tissue. The colon mucosa is composed of thick and complete epithelial cells, which promote colon movement by regulating the water absorption of the intestinal cavity [2]. HSDF intervention significantly alleviated the thinning of the colon wall in mice, normalized the arrangement of intestinal villi, reduced the infiltration of inflammatory cells, and restored the secretion of mucosal goblet cells and mucus, indicating that HSDF could improve the intestinal tissue damage in mice with constipation. Certain studies have shown that Polysaccharide Spirulina platensis has a therapeutic effect on mice with constipation as it improves AChE (acetylcholinesterase) activity and repairs damaged intestinal villi [36], which is consistent with the results of this study. Cytokines include IL-6 and TNF-α, which are closely related to the intestinal immunity. IL-6 aids in cell activation, differentiation, and maturation. TNF-α has tumor necrosis activity [37]; IL-1β attracts neutrophils and causes the release of inflammatory mediators, which promotes the expression of vascular leukocyte adhesion molecules and induces inflammatory responses [38]. In this study, HSDF reduced the expression of inflammatory factors and alleviated colitis in mice, suggesting that HSDF can alleviate constipation by reducing intestinal inflammation, which is consistent with the results obtained by Kim [39].

Studies have confirmed that a large amount of AQP is distributed in the intestinal mucosa and plays a crucial role in intestinal absorption, secretion, and regulation of water metabolism by mediating the transmembrane transport of water molecules. Compared with the M group, the active treatment in the HS group, LHDF group, and HHDF group promoted the increase level of hormone VIP [40], and the expression levels of AQP3 and AQP8 proteins were high. Among them, the HHDF group had the highest effect, with an increase of 52.67% and 164.54%, respectively, which can promote the absorption of water in colon epithelial cells and alleviate constipation [41]; The active treatment in the HS group, LHDF group, and HHDF group inhibited the expression of AQP9 protein and increase intestinal peristalsis frequency, and the HHDF group had the best inhibitory effect.

SCFAs are important metabolic energy sources in the human body. Therefore, constipation also has abnormal SCFAs. SCFAs can stimulate the contraction of colonic smooth muscle to accelerate intestinal propulsion, reduce pH and inhibit pathogenic microorganisms, stabilize intestinal health, alleviate inflammation, maintain the integrity of the intestinal barrier, and promote defecation [42]. SCFAs mainly include acetic, propionic, isobutyric, butyric, isovaleric, and valeric acid. The acetic, protonic, and isobutyric acids in the colon account for 90–95% of the total SCFAs, which are derived from the escaped carbohydrate and protein and absorbed in the small intestine during the fermentation and digestion of the intestinal flora [43]. Isomeric acid in SCFAs can enhance the intestinal barrier function by regulating the expression of tight junction proteins [44]. Propionic acid is an important energy substrate of colon cells and acetic acid is an important energy source of the liver [45]. Maren soft capsule was found to have the ability to regulate the metabolic pathway of SCFAs in the colon contents of STC rats by increasing the contents of acetic, propionic, and isobutyric acids, thus relieving constipation symptoms [46]. We found that compared with group M, the LHDF and HHDF groups had increased levels of acetic, propionic, butyric, isovaleric, and valeric acid. Interestingly, the SCFA increase in the HHDF group exceeded that in the HS maren soft capsule group. The intervention of HSDF can restore SCFAs reduced by loperamide hydrochloride, maintain the intestinal pH and health of the intestinal wall, and has remarkable application potential in relieving constipation. Compared with group M, the LHDF and HHDF groups had increased levels of acetic, propionic, butyric, isovaleric, and valeric acids. Interestingly, the increase in SCFAs in the HHDF group exceeded that in the HS maren soft capsule group. The intervention of HSDF can restore the SCFAs reduced by loperamide hydrochloride, maintain the intestinal pH and health of the intestinal wall, and has remarkable application potential in relieving constipation. Definitly, the dietary fiber was proved to promote the secretion of short-chain fatty acids such as acetic acid and propionic acid, increase the proliferation of probiotics, improve the intestinal environment, and thus alleviate constipation [18]. And Yang Guang et al. confirmed that hawthorn dietary fiber has the effect of moistening the intestines and promoting bowel movements when studying how hawthorn dietary fiber alleviates intestinal constipation in mice [47]. Many studies have shown that after dietary fiber supplementation, the intestinal microflora ferment indigestible polysaccharides, produce SCFAs, and reduce the intestinal pH value, which is consistent with our research results [48].

Abnormal SCFAs along with an imbalance in the intestinal flora can indicate constipation in patients, mainly manifested as a decreased abundance of Bifidobacterium, Lactobacillaceae, and Fusobacterium in the colonic mucosa. The intestinal flora form a biological barrier on the intestinal mucosa through adhesion or combination. High diversity in the intestinal flora indicates a healthy intestinal environment. Studies have shown that polysaccharides play an important role in regulating intestinal flora, immunosuppression, and antioxidation [49,50]. Undigested dietary fiber is regarded as the nutrient source of intestinal flora and play a key role in host health [51]. Our results showed that HSDF improved the intestinal flora diversity and flora species diversity of mice with constipation. In addition, the high-dose group had increased abundance of Firmicutes, which mainly produce butyric acid and aid in repairing the intestinal mucosa and improving the intestinal barrier, which might be another mechanism of HSDF for relieving symptoms of constipation.

Previous studies have reported that the abundance changes in Alistipes and Rikenellaceae are related to gastrointestinal function and significantly affect the intestinal motility, colonic mucin secretion, or intracolonic transport time [3]. The abundance of these two bacteria in the colon of mice with constipation was significantly reduced. Alloprevotella is a strictly anaerobic intestinal bacterium that can degrade mucins to produce propanoic acid and improve the intestinal barrier. HSDF could affect the secretion of intestinal microbial metabolite SCFAs and bile acids by regulating the abundance of Lactobacillaceae, Alistipes, Rikenellaceae, and Alloprevotella in the intestine, regulate the conduction of intestinal signaling pathways, and stimulate intestinal peristalsis, thereby relieving the symptoms of constipation.

## 5. Conclusions

This study found that galacturonic acid is the monosaccharide component of HSDF extracted via the microwave enzymatic method, and has obvious polysaccharide features, a loose structure, coexistence of crystalline and amorphous regions, and structural features that relieve constipation. HSDF alleviates loperamide hydrochloride-induced functional constipation by regulating gastrointestinal hormones, reducing colon inflammation, improving the integrity of the intestinal barrier, balancing the intestinal flora, enhancing the stability of the intestinal flora, improving the content of metabolite SCFAs, and regulating the AQP water ion pathway. HSDF can increase intestinal probiotics, regulate the imbalance of intestinal flora, and alleviate the symptoms of constipation more gently and efficiently.

HSDF might become a safe and effective functional food raw material for relieving constipation. It can improve the environmental burden and increase the economic value and has remarkable application prospects. Notably, we have verified for the first time that HSDF can alleviate constipation by regulating the abundance and species of intestinal flora, regulate intestinal pH by affecting the metabolism of SCFAs, protect intestinal health, and alleviate the symptoms of constipation induced by loperamide hydrochloride. These results provide a reference for follow-up researchers. However, research on the 5-hydroxytryptamine (5-HT) receptor pathway in the colon of mice, the regulating pathway of intestinal metabolism to alleviate constipation symptoms, and the efficacy of different kinds of dietary fiber and their effects on the human body should be studied further.

## Figures and Tables

**Table 1:** Particle size analysis of soluble dietary fiber in hawthorn residue.

Distribution Name	Mn (Daltons)	Mw (Daltons)	MP (Daltons)	Mz (Daltons)	Polydispersity
1	14,787	17,973	13,587	22,127	1.215433
2	2744	3381	1558	4219	1.232293

Number-average molecular weight: Mn; weight-average molecular weight: Mw; highest-peak molecular weight *p*: Mp; Z-average molecular weight: Mz; multiple dispersion coefficient: Polydispersity, *n* = 3.

**Table 2:** Analysis of monosaccharide composition in HSDF.

Monosaccharide Composition	Monosaccharide Content/(μg/mg)
d-mannose	2.07 ± 0.08
l-rhamnose monohydrate	24.67 ± 1.02
l-arabinose	113.22 ± 3.12
d-galactose	39.45 ± 1.13
Glucose	42.83 ± 1.32
d-xylose	7.20 ± 0.23
d-mannose	3.12 ± 0.16
d-galacturonic acid	306.07 ± 6.35
d-glucuronic acid	1.55 ± 0.05
Total	540.18 ± 6.58

Number of samples tested: *n* = 6.

**Table 3:** The effect of HSDF on hormone levels in various groups of mice.

Groups	MTL (pg/mL)	GAS (pg/mL)	SP (pg/mL)	VIP (pg/mL)	SS (pg/mL)	NO (μg/mL)	MDA (nmoL/mL)
C	83.72 ± 7.18	6.61 ± 0.79	108.35 ± 5.63	89.54 ± 4.07	45.24 ± 3.61	8.00 ± 0.09	27.61 ± 0.87
M	52.14 ± 11.49 **	4.28 ± 0.09 **	96 ± 3.81 *	31.59 ± 2.72 **	102.66 ± 5.37 **	9.10 ± 0.28 **	53.14 ± 1.59 **
HS	62.14 ± 4.53	6.21 ± 0.44	91.98 ± 3.81	58.51 ± 3.56 ^##^	58.91 ± 3.31 ^##^	5.34 ± 2.40 ^##^	26.72 ± 1.51 ^##^
LHDF	72.49 ± 4.25 ^##^	5.18 ± 0.26 ^##^	135.58 ± 12.50 ^##^	44.67 ± 3.95 ^#^	94.74 ± 5.65 ^#^	8.44 ± 0.75 ^#^	46.37 ± 1.84 ^##^
HHDF	85.85 ± 2.45 ^##ss^	6.00 ± 0.88 ^##ss^	142.98 ± 14.28 ^##s^	73.72 ± 4.24 ^##ss^	49.62 ± 2.33 ^##ss^	6.20 ± 1.00 ^##ss^	27.02 ± 0.95 ^##ss^

Values are shown as mean ± SD (*n* = 5), *p* < 0.05. We used the unpaired two-tailed Student’s *t*-test with Bonferroni correction. Compared with group C: ** *p* < 0.01, * *p* < 0.05. Compared with group M: ^##^ *p* < 0.01, ^#^ *p* < 0.05.Compared with LHDF: ^ss^ < 0.01, ^s^ < 0.05. MTL, motilin, GAS, gastrin, SP, substance P; VIP, vasoactive intestinal peptide; SS, somatostatin; NO, nitric oxide; MDA, malondialdehyde; C, blank control group; HHDF, high-dose group (2.0 g/kg bw); HS, maren soft capsule positive control group (0.4 g/kg bw); LHDF, low-dose group (1.0 g/kg bw); M, loperamide hydrochloride model group (10 mg/kg bw).

## Data Availability

The original contributions presented in the study are included in the article, further inquiries can be directed to the corresponding authors.
